# miR-146a is essential for lipopolysaccharide (LPS)-induced cross-tolerance against kidney ischemia/reperfusion injury in mice

**DOI:** 10.1038/srep27091

**Published:** 2016-06-02

**Authors:** Yan Dai, Ping Jia, Yi Fang, Hong Liu, Xiaoyan Jiao, John C. He, Xiaoqiang Ding

**Affiliations:** 1 Division of Nephrology, Zhongshan Hospital, Fudan University, Shanghai, China; 2 Kidney and Dialysis Institute of Shanghai, Shanghai, China; 3 Kidney and Blood Purification Laboratory of Shanghai, Shanghai, China; 4 Department of Medicine/Nephrology, Icahn School of Medicine at Mount Sinai, New York, New York, USA; 5 Kidney Section, James J Peter Veteran Administration Medical Center at Bronx, NY, United States

## Abstract

MicroRNA-146a is one of most important microRNAs involved in development of endotoxin tolerance via (toll-like receptors) TLRs/ NF-κB pathway. In this study, we sought to identify the mechanistic role of miR-146a in mediating the protective effect of lipopolysaccharide (LPS) pretreatment on kidney ischemia/reperfusion injury. A locked nucleic acid–modified anti-miR-146a given before LPS treatment knocked down miR-146a expression and completely negated LPS-mediated protection against kidney ischemia/reperfusion injury. Knockdown of miR-146a resulted in significantly higher histopathological scores for tubular damage, expression of proinflammatory cytokines and chemokines, and neutrophil and macrophage infiltration. Furthermore, knockdown of miR-146a greatly up-regulated the protein levels of IL-1 receptor-associated kinase (IRAK-1) and tumor-necrosis factor (TNF) receptor-associated factor 6 (TRAF6), which are known target genes of miR-146a, leading to activation of NF-κB. Finally, elevation of nuclear translocation of NF-κB p65/p50 and caspase-3 expression, degradation of cytosolic IkBα and BcL-xL, and substantially exacerbation of tubular cell apoptosis were inversely correlated with miR-146a expression. Taken together, our results identify that miR146a exerts a kidney protective effect through negative regulation of acute inflammatory response by suppressing NF-κB activation and proinflammatory genes expression.

Acute kidney injury (AKI) is a major clinical problem with a high degree of morbidity and mortality^1^. Kidney ischemia/reperfusion (I/R) injury is the major cause of acute kidney injury after major surgery or kidney transplantation^2^,^3^. I/R injury may activate innate immunity by endogenous ligands through the engagement of Toll-like receptors (TLRs), particularly TLR4 and TLR2^4^,^5^,^6^. TLR4 expressed within the kidney is a potential mediator of innate activation and inflammation^7^. Uncontrolled inflammation results in extensive tissue damage, and therefore this important inflammatory reaction must be tightly regulated; this regulatory response is seen in the manifestation of endotoxin tolerance^8^,^9^.

Endotoxin tolerance is claimed to be a specific phenomenon, where animals injected with subtoxic LPS dose show an increased survival rate against inflammation damage *in vivo* and endotoxin-primed cells show reduced responsiveness toward repeated endotoxin stimulation *in vitro*^10^,^11^,^12^. Cross-tolerance, which means following endotoxin exposure, endotoxin-primed cells are hyporesponsive to stimulation with other TLR ligands has also been described^13^. LPS is a classical ligand for TLR4 and mediates TLR4/MyD88-dependent signal transduction to activate NF-κB, leading to an increase in inflammatory cytokine expression such as IL-1β and TNF-α^14^,^15^,^16^. However, sepsis/endotoxin and inflammation are frequently observed complications during kidney I/R injury. Endotoxin can play a role in kidney I/R injury induced during clinical procedures. Thus, preconditional LPS treatment provokes cross-tolerance and protects kidney against a subsequent, sustained ischemic result^17^.

MicroRNAs (miRNAs), a family of small noncoding RNAs which regulate gene expression by base-pairing to the 3′ untranslated region of their target genes, have recently emerged as a novel class of posttranscriptional regulators in the immune system, including endotoxin tolerance^18^,^19^,^20^. Previously, a number of different miRNA (miR-146a, miR-98, miR-155) have been found to be involved in regulation of endotoxin tolerance^21^. Among them, miR-146a attracted our attention: previous studies show that LPS-induced up-regulation of miR-146a is indeed NF-κB dependent^22^; Importantly, two key adapter molecules in the TLR4/NF-κB pathway, TRAF6 and IRAK-1 are identified as direct targets of miR-146a, and they also promote inflammation stimulated by proinflammatory cytokines including TNF-a^23^. Above finding suggests the role of miR-146a in TLR4/ NF-κB signaling through a negative feedback regulatory loop, which can suppress acute inflammatory response^24^. Experiments with THP-1 cells have shown that miR-146a participates in transcriptional and translational modifications associated with gene reprogramming during induction of endotoxin tolerance^13^,^25^. This leads to the speculation that increased miR-146a expression during an LPS-primed state might play a part in a negative feedback pathway for other ligand-TLRs interactions such as TLR4/Myd88-dependent signaling implicated in a mouse model of ischemic AKI^26^. Additionally, increased miR-146a expression also has been confirmed during kidney I/R injury^27^. Thus, we focused on miR-146a for further study. In this study, with highly effective *in vivo* knockdown of miR-146a, we explored the role of miR-146a in mediating the protective effect of LPS pretreatment on kidney ischemia/reperfusion injury.

## Results

### Knockdown of miR-146a negates the kidney protection conferred by LPS

**Figure 1:**
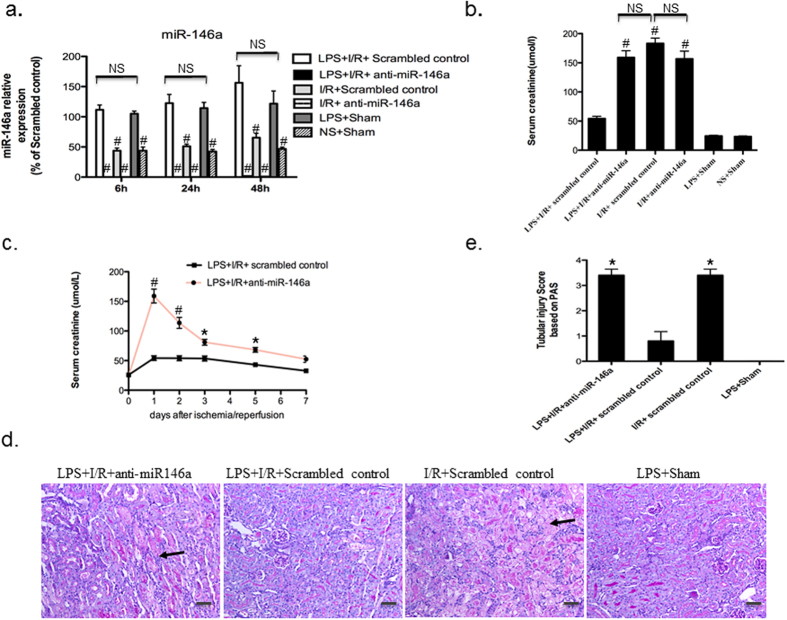
LPS pretreatment protects mice from kidney I/R injury, which is negated by knockdown of miR-146a. (**a**) Locked nucleic acid (LNA) anti-miR-146a (10 mg/kg) or anti-scrambled was administrated 2 hour prior to LPS injection. At 24 h after exposure to either normal saline (NS) or lipopolysaccharide (LPS; 3 mg/kg, intraperitoneal injection), mice were subjected to kidney ischemia surgery. miR-146a expression was examined and normalized to u6 at 6, 24, 48 h after kidney I/R by TaqMan-based real-time PCR, fold changes were calculated against the mean value of LPS + Sham group at each time point. (n = 6 mice per group for each time point. ^#^*P* < 0.001, vs. LPS + I/R + Scrambled control, respectively. NS, not significant). (**b**) Serum creatinine was measured and data was expressed as mean ± SEM at 24 h after kidney I/R injury. (n = 6 mice per group, ^#^*P* < 0.001 vs. LPS + I/R + Scrambled control). (**c**) Time course of serum creatinine levels after I/R in mice exposed to LPS with or without miR-146a knockdown. (n = 4 mice per group for each time point, *P < 0.05, ^#^*P* < 0.001 vs. LPS + I/R + Scrambled control). (**d**) Representative periodic acid–Schiff (PAS)-stained kidney sections from mice receiving anti-miR-146a or anti-scrambled oligonucleotides at 24 h after pretreatment with either vehicle + I/R or LPS + I/R procedures (original magnification x200, Bar = 50 um. The black arrow indicates damaged tubules). (**e**) Abnormalities based on PAS-stained sections were graded by a semiquantitative histomorphological scoring system from 0 to 4. *P < 0.05 vs. LPS + I/R + Scrambled control.

To determine the functional role of miR-146a in the kidney protection conferred by LPS pretreatment, locked nucleic acid (LNA)–modified anti-scrambled or anti-miR-146a oligonucleotides (10 mg/kg) were administered to mice through the tail vein just 2 hour prior to LPS (3 mg/kg, ip) or vehicle treatment. At 24 h after LPS injection, the kidneys were subjected to I/R procedures and then harvested. As a first step to investigate the role of miR-146a in cross-tolerance, we measured its expression profile in the kidney activated with LPS. As shown in Fig. 1a, the miR-146a levels of LPS + I/R + Scrambled control group increased upon LPS stimulation at all time points compared with LPS + I/R + anti-miR-146a group and I/R + anti-miR-146a group (P < 0.001), whereas there was no change compared with LPS + Sham group (P > 0.05). In contrast, miR-146a levels in LPS + I/R + anti-miR-146a and I/R + anti-miR-146a group were markedly decreased after its knockdown (Fig. 1a). To analyze the role of miR-146a in injury and repair responses following kidney I/R, we studied additional time course of miR-146a expression and compared those of miR-21, which is also known to contribute to I/R injury. MicroRNA-21 was immediately up-regulated following I/R injury compared to sham samples at day 0 with a peak at day3. However, miR-146a was up-regulated after day3 and achieved its peak at day7 (Supplementary Figure 1a). Serum creatinine measured at 24 h after I/R injury was also significantly elevated in LPS + I/R + anti-miR-146a group and I/R + Scrambled control group as compared to LPS + I/R + Scrambled control group (159.1 ± 11.6 umol/l vs. 183.2 ± 9.1 umol/l vs. 54.25 ± 3.96 umol/l, P < 0.001, Fig. 1b). There was no statistical difference in serum creatinine levels between LPS + Sham and LPS + I/R + Scrambled control groups. However, both I/R + anti-miR-146a group and I/R + Scrambled control group mice displayed a similar elevation in serum creatinine (183.2 ± 9.1 umol/l vs. 156.8 ± 13.32 umol/l). To confirm the robustness of the observed effect of anti-miR-146a, we measured serum creatinine levels at 0, 1, 2, 3, 5, or 7 days following the I/R injury in additional groups of mice with LPS + I/R + anti-miR-146a and LPS + I/R + Scrambled control. Mice treated with LPS + I/R + Scrambled control were largely protected from the I/R injury (Fig. 1c).

The functional data correlated with histological evidence in periodic acid-Schiff-stained sections. As shown in Fig. 1d and Supplementary Figure 1c, mice receiving LPS + I/R + anti-miR-146a exhibited severe tubular damage, as evidenced by widespread tubular necrosis, loss of the brush border, cast formation, cellular swelling and serious inflammatory infiltration at the corticomedullary junction, maximal at 24 h, whereas LPS + I/R + Scrambled control group showed significantly less tubular damage and mild cellular swelling as compared with LPS + I/R + anti-miR-146a group and I/R + Scrambled control group at 24 h after I/R injury (Fig. 1e). LPS + Sham group incurred no tubular injury. It is important to note that both I/R + anti-miR-146a group and I/R + Scrambled control group mice showed a similar morphological damage score (P > 0.05, Supplementary Figure 1d). These results suggest that LPS pretreatment increases miR-146a expression and protects mice from kidney I/R injury, which is negated by knockdown of miR-146a *in vivo*.

### Induction of miR-146a in mouse kidneys by LPS suppresses I/R injury-induced inflammatory cytokine production

**Figure 2:**
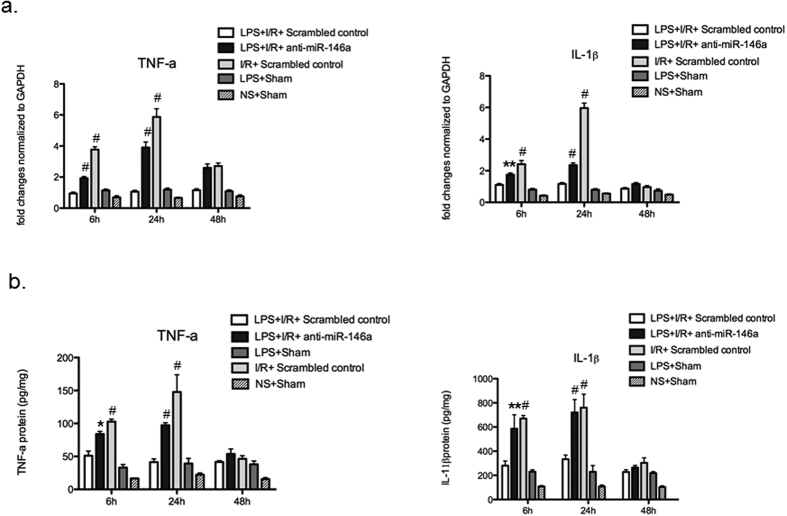
Proinflammatory cytokine (IL-1β and TNF-α) production in the LPS-challenged kidney following I/R injury. (**a**) mRNA of IL-1β and TNF-α in the kidney medulla was determined by real-time PCR and was normalized to GAPDH at 6, 24, 48 h after kidney I/R. (n = 6 mice per group, **P < 0.01, ^#^*P* < 0.001 vs. LPS + I/R + Scrambled control). (**b**) IL-1β and TNF-α protein levels at each time point were evaluated by Enzyme-linked immunosorbent assay (ELISA) at each time point. n = 6 mice/group, *P < 0.05, **P < 0.01, ^#^*P* < 0.001 vs. LPS + I/R + Scrambled control). All data were expressed as mean ± SEM.

**Figure 3:**
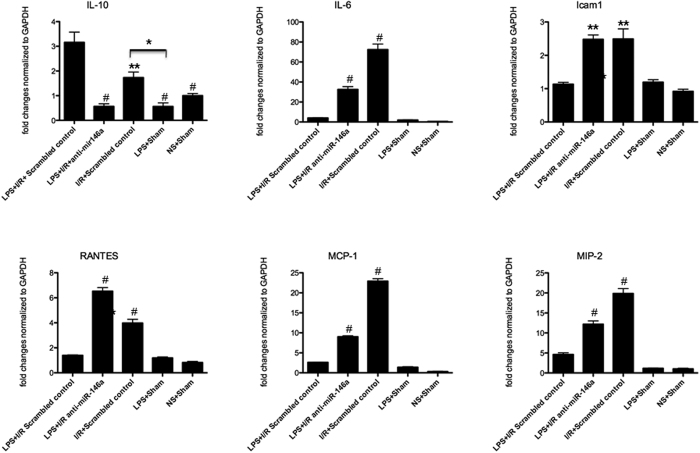
Inflammatory mediators mRNA profile in the LPS-challenged kidney following I/R injury. Kidney expression of the proinflammatory cytokines and chemokines were quantified by real-time PCR at 24 h after kidney I/R injury. (n = 6 mice per group, *P < 0.05, **P < 0.01, ^#^*P* < 0.001 vs. LPS + I/R + Scrambled control).

It has been reported that NF-κB-dependent proinflammatory cytokines, such as IL-1β, TNF-α, IL-6, are down-regulated, whereas anti-inflammatory cytokines, such as IL-10, TGF- β, and IL-1RA, are up-regulated in endotoxin tolerance models^21^. IL-1β and TNF-α mRNAs examined by real-time PCR in LPS + I/R + anti-miR-146a group was significantly higher than LPS + I/R + Scrambled control group at 6 h and 24 h after I/R injury, peaking at 24 h (Fig. 2a), then declined at 48 h. Analysis of protein levels of IL-1β and TNF-α by Enzyme-linked immunosorbent assay (ELISA) showed levels consistent with the mRNA data, and induction of IL-1β and TNF-α in LPS + I/R + anti-miR-146a group was significant elevated compared with LPS + I/R + Scrambled control group (Fig. 2b). As shown in Fig. 3, we also measured the mRNA levels of cytokines (IL-10 and IL-6). Lower mRNA level of IL-10 was detected in mice receiving LPS + I/R + anti-miR-146a group while significantly increased in LPS + I/R + Scrambled control group. We found a slightly increase of IL-10 mRNA in I/R + Scrambled control group. For IL-6, mRNA levels were strongly up-regulated in LPS + I/R + anti-miR-146a group at 24 h after kidney I/R injury, and that of I/R + Scrambled control group also increased but to a greater extent compared with LPS + I/R + Scrambled control group (Fig. 3).

Kidney expression of the chemokines (macrophage inflammatory protein-2, MIP-2, monocyte chemoattractant protein-1, MCP-1 and RANTES) was significantly higher in mice receiving LPS + I/R + anti-miR-146a and I/R + Scrambled control group, compared with LPS + I/R + Scrambled control group (Fig. 3). Similarly, significant differences were found for intercellular adhesion molecule-1 (Icam1), a key regulator of leukocyte adhesion and transendothelial migration (Fig. 3). There was no statistically difference between LPS + I/R + Scrambled control group and LPS + Sham group (P > 0.05). However, there was no statistically difference between I/R + Scrambled control group and I/R + anti-miR-146a group mice at 24 h after reperfusion (6 h and 48 h data not shown, P > 0.05, Supplementary Figure 1b). Consistent with the histological data, knockdown of miR-146a in mice receiving only I/R procedures showed a similar increase of the kidney proinflammatory cytokines production after reperfusion. Taken together, these results indicate that miR-146a contributes to LPS induced cross-tolerance against kidney I/R injury by inhibiting proinflammatory pathways.

### Knockdown of miR-146a promotes inflammatory cell infiltration and apoptosis

**Figure 4:**
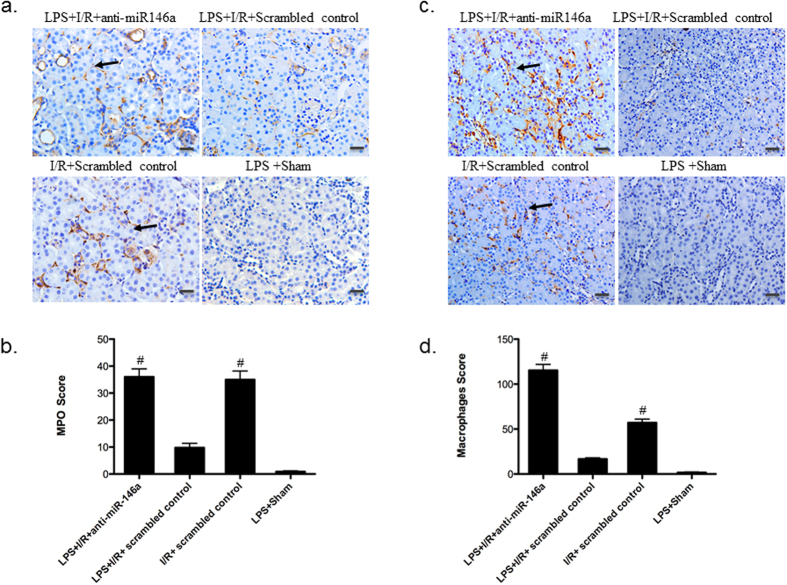
Inflammatory cell infiltration. (**a**) Representative kidney sections immunostained for myeloperoxidase (MPO) to show polymorphonuclear (PMN) leukocyte infiltration (original magnification x200, Bar = 50 um, the black arrow indicates positive area). (**b**) PMN infiltration was scored in mouse kidney. (n = 6 mice per group, ^#^*P* < 0.001, vs. LPS + I/R + Scrambled control). (**c**) Representative kidney sections immunostained for CD68 to show macrophage accumulation within the interstitium of the kidney (original magnification x200, Bar = 50 um). (**d**) Analysis of macrophage infiltration in mouse kidney. (n = 6 mice per group, ^#^*P* < 0.001, vs. LPS + I/R + Scrambled control). All data were expressed as mean ± SEM.

Inflammatory cells have been shown to have a critical role in the pathogenesis of I/R injury^28^. To further characterize the role of miR-146a in the inflammatory cells, immunoperoxidase labeling for CD68 (a macrophage marker) and myeloperoxidase (MPO) (a neutrophil marker) on paraffin-embedded kidney sections were performed at 24 h after I/R injury. Polymorphonuclear (PMN) leukocyte infiltration was significantly less in LPS + I/R + Scrambled control group compared to that of LPS + I/R + anti-miR-146a group and I/R + Scrambled control group (Fig. 4a,b). Consistent with the neutrophil accumulation, staining of CD68 localized in the outer medulla showed that LPS + I/R + anti-miR-146a group induced a progressive increase in interstitial at 24 h after I/R injury. Compared with LPS + I/R + anti-miR-146a group or I/R + Scrambled control group, LPS + I/R + Scrambled control had significantly less interstitial macrophages during I/R injury (Fig. 4c,d).

**Figure 5:**
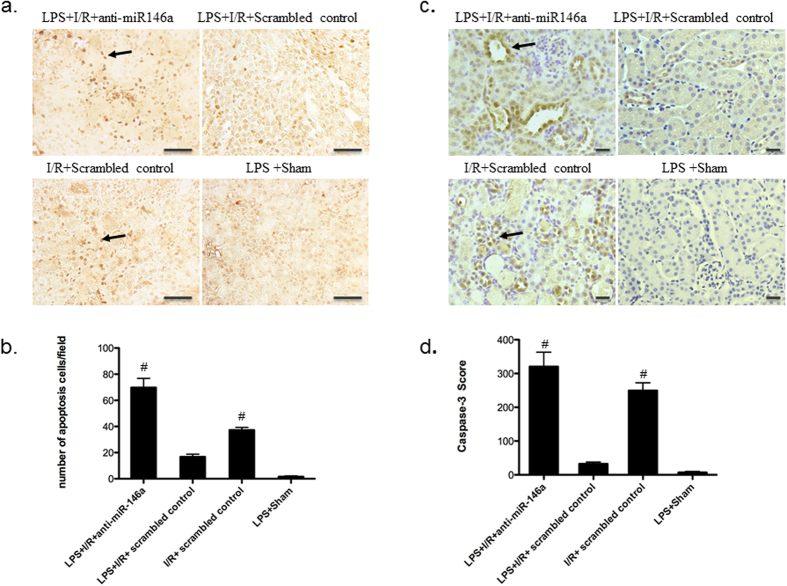
Inhibiting miR-146a activity in the LPS-challenged kidney exacerbates apoptosis during I/R injury. (**a**) Terminal deoxynucleotidyl transferase-mediated dUTP nick-end labeling (TUNEL)-positive cells in kidney sections, photographed at original magnification x400, Bar = 50 um. The arrow indicates apoptotic tubular cell. (**b**) Mean value of TUNEL-positive cells in kidney sections. Knockdown of miR-146a increased kidney cell apoptosis. (n = 6 mice per group, ^#^*P* < 0.001, vs. LPS + I/R + Scrambled control). (**c**) Representative micrographs showing immunohistochemical staining for cleaved caspase-3 among different groups as indicated. (original magnification x200 Bar = 50 um). (**d**) Analysis of cleaved caspase-3 accumulation in mouse kidney. (n = 6 mice per group, ^#^*P* < 0.001, vs. LPS + I/R + Scrambled control). All data were expressed as mean ± SEM.

Tubular cell apoptosis has been shown to contribute to the pathogenesis of ischemic kidney injury^29^; we therefore investigated the role of miR146a in ischemia–reperfusion induced tubular epithelial cell apoptosis. Our results showed that there was a significant increase in the number of tubular apoptotic cells as assessed by terminal transferase dUTP nickend labeling (TUNEL) in LPS + I/R + anti-miR-146a group (Fig. 5a,b). LPS pretreatment attenuated kidney tubular cell apoptosis 24 h after I/R (n = 6 mice per group), suggesting a critical role of miR-146a in LPS mediated renoprotection. Caspase-3 is the final effector caspase that mediates apoptotic cell death. As such, immunostaining demonstrated that cleaved caspase-3 protein expressed by tubular epithelial cells (TECs) particularly at the corticomedullary junction. The amount of caspase-3 detected in LPS + I/R + Scrambled control group was substantially less compared with that seen in LPS + I/R + anti-miR-146a and I/R + Scrambled control group (Fig. 5c,d).

### Knockdown of miR-146a enhances TRAF6 and IRAK-1 protein expression

MicroRNA-146a down-regulates the expression of IRAK-1 and TRAF-6 signaling molecules involved in LPS/TLR4 mediated activation of innate immune response^24^. To further demonstrate the expression of known miR-146a target genes in kidney medulla, TRAF6 and IRAK-1, were determined by real-time PCR and western blot analysis in LPS or normal saline treated animals in a time course (6 h, 24 h, 48 h) following I/R injury.

**Figure 6:**
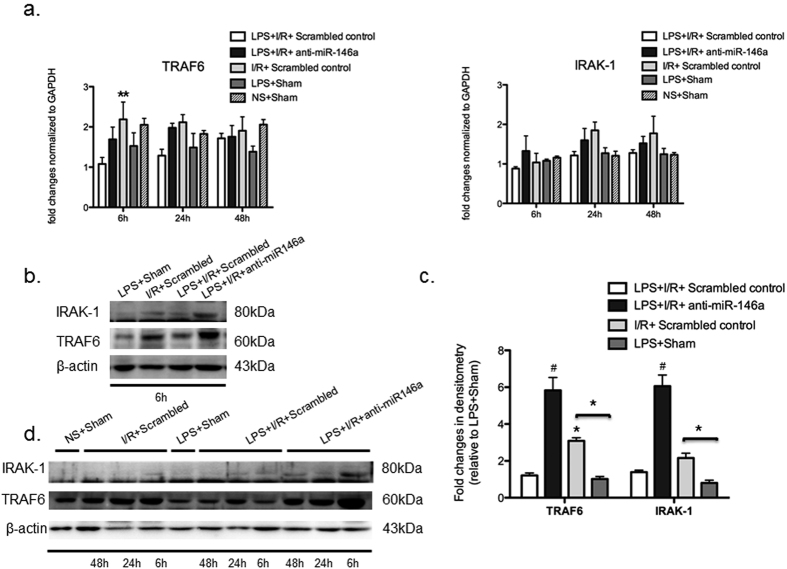
Knockdown of miR-146a promotes TRAF6 and IRAK-1 protein expression. LNA-anti-miR-146a or anti-scrambled oligonucleotides (10 m/kg) were administered 2 h before LPS treatment. After 24 h LPS exposure, the kidneys were subjected to I/R or Sham procedures followed by 6 h, 24 h, 48 h of reperfusion and then harvested. (**a**) mRNA of TRAF6 and IRAK-1 in the kidney medulla was determined by real-time RT-PCR and normalized to GAPDH at all time points. (n = 6 mice per group, *P* > 0.05 vs. LPS + I/R + Scrambled control group). (**b**) Kidney lysates of 6 h groups were subject to Western blot analysis for TRAF6 and IRAK-1. (**c**) The Western blots from all 6 h experiments were quantified by densitometry analysis. The ratios of TRAF6/ β-actin and IRAK-1/ β-actin were calculated; the fold changes relative to LPS + Sham protein are shown. (n = 6 mice per group, *P < 0.05, ^#^*P* < 0.001, vs. LPS + I/R + Scrambled control group). (**d**) Time course of TRAF6 and IRAK-1 expression was confirmed by Western-blot in the LPS-challenged kidney following I/R injury.

As shown in Fig. 6a, mRNA transcript levels of TRAF6 and IRAK-1 were not markedly different between mice with and without miR-146a knockdown at all time points after I/R injury. There was a slightly increase of mRNA levels of TRAF6 in I/R + Scrambled control group at 6 h time points. In contrast, a significant increase of TRAF6 protein levels occurred at 6 h (Fig. 6b,c) and remained at later time up to 48 h (Fig. 6d) from LPS + I/R + anti-miR-146a and I/R + Scrambled control mice but not from LPS + I/R + Scrambled control mice. However, induction of IRAK-1 was also observed from LPS + I/R + anti-miR-146a mice at 6 h and sharply reduced at 24 h, whereas mice in LPS + I/R + Scrambled control group expressed negligible levels of IRAK-1 at all time points (Fig. 6d). Our data confirmed that miR-146a mediated down-regulation of TLR4/ NF-κB signaling via targeting TRAF6 and IRAK-1 in LPS-challenged kidney following I/R injury.

### Knockdown of miR-146a activates nuclear translocation of NF-κB p65/p50

**Figure 7:**
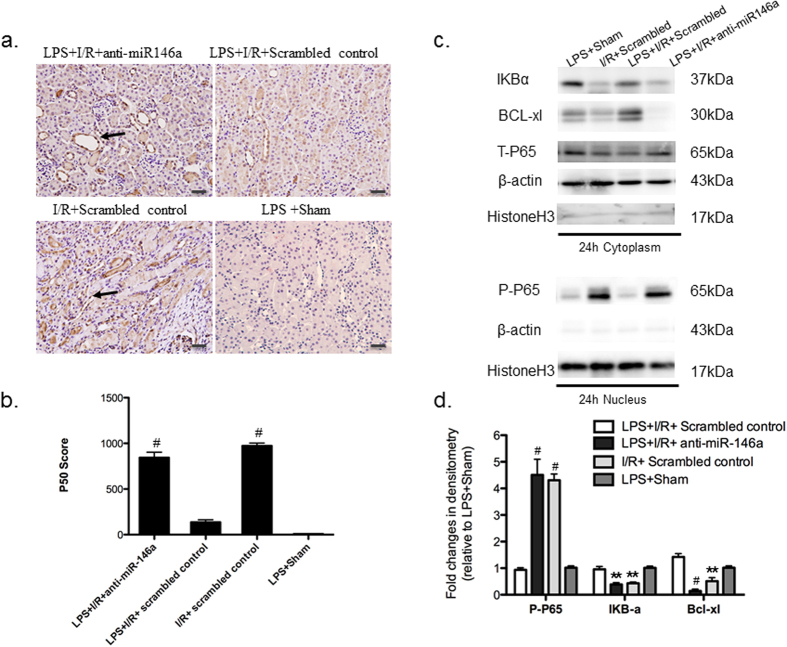
The link of miR-146a and NF-κB activation. (**a**) Immunohistochemistry shows positive nuclear NF-κB p50 staining 24 h after I/R in mice exposed to LPS with or without miR-146a knockdown (original magnification x200, Bar = 50 um) (**b**) Nuclear NF-κB p50 expression was scored in mouse kidney. (n = 6 mice per group, ^#^P < 0.001, vs. LPS + I/R + Scrambled control group). The specificity of the staining was further demonstrated by the absence of signals in LPS + Sham group (**c**) Kidney lysates of 24 h groups were probed with specific antibody against phosphorylated P65 (p-p65) (nuclear extracts), and IkBα, BcL-xL, T-P65 (cytosolic extracts). Co-detection of Histone H3 (nuclear) and β-actin (cytoplasm) were performed to assess equal loading. A significant increase in nuclear NF-κB p65 expression, cytosolic IkBα and BcL-xL degradation at 24 h was observed. (**d**) The Western blots from all experiments were quantified by densitometry analysis. The ratios of phosphor-protein to total protein for p65 were calculated. The fold changes relative to LPS + Sham protein are shown. (n = 3, ^#^*P* < 0.001, **P < 0.01, vs. LPS + I/R + Scrambled control group).

TLR4/ NF-κB pathway is involved in the mechanism of LPS induced endotoxin and cross-tolerance^21^. Signaling through MyD88 leads to phosphorylation-mediated degradation of inhibitors of NF-κB (IκBs), nuclear translocation of NF-κB p65/p50 and transcriptional activation of proinflammatory genes^31^. To determine the link of miR-146a and NF-κB activation, we measured protein levels of phosphorylated P65 (p-p65) in nuclear extracts and p65, IkBα in cytoplasm at 6, 24 and 48 h after reperfusion. To confirm the nuclear translocation of NF-κB p50, immunohistochemical localization of p50 was carried out at 24 h after I/R injury (Fig. 7a,b, 6 h and 48 h data not shown). LPS pretreatment completely blocked the nuclear localization of p50 induced by I/R injury, which can be reversed by knockdown of miR-146a. Likewise, knockdown of miR-146a increased the expression of nuclear NF-κB p65. As compared to the mice in LPS + I/R + Scrambled control group, mice in the LPS + I/R + anti-miR-146a group had approximately five-fold increase of nuclear NF-κB p65 expression at 24 h (4.8-fold; Fig. 7c,d) and at 6 h (4.7-fold; Supplementary Figure 2a,c). A 2.5-fold increase was also observed at 48 h (Supplementary Figure 2b,d). Mice in the I/R + Scrambled control group showed a similar elevation of NF-κB p65 nuclear translocation at all time points. Either normal saline (NS) or LPS treated Sham scrambled control mice showed the same NF-κB activity baseline (data not shown). Furthermore, the increased NF-κB activation was accompanied by a simultaneous decrease in cytosolic IkBα, an inhibitory protein that prevents translocation of NF-κB into the nucleus (Fig. 7c,d, Supplementary Figure 2).

The balance between pro- and anti-apoptotic forces determines the fate of tubule cells underwent an ischemic insult, as evidenced by studies both in rodents^32^,^33^ and in kidney epithelial cells *in vitro*^34^. As such, we examined the effect of miR-146a knockdown on Bcl-extralarge (BcL-xL) protein expression in the kidney. As results indicated above, there is a general inverse correlation between miR-146a expression and kidney tubular cell apoptosis assessed by TUNEL staining. This observation correlated with decreased protein level of antiapoptotic BcL-xL, which can be reversed in LPS + I/R + Scrambled control group (Fig. 7c, Supplementary Figure 2a,b). Collectively, these findings support the hypothesis that elevation of nuclear translocation of NF-κBp65/p50, degradation of cytosolic IkBα and BcL-xL, and substantially exacerbation of tubular cell apoptosis are inversely associated with miR-146a expression. MicoRNA-146a can function as a key regulator in the development of LPS-induced cross-tolerance by inhibition of NF-κB.

## Discussion

Although miR-146a has been recognized to function in the endotoxin tolerance for a long time^12^,^13^,^25^, the mechanistic role of miR-146a underlying LPS-induced cross-tolerance against kidney I/R injury remains unclear. The present study has revealed a novel role of miR146a *in vivo* that induction of miR146a by LPS pretreatment protects kidney from I/R injury. We postulate that miR-146a could function as a key regulator in the development of LPS-induced cross-tolerance through one of three potential mechanisms: (1) elevation of nuclear translocation of NF-κBp65/p50 and substantially exacerbation of tubular cell apoptosis are inversely correlated with miR-146a expression. (2) miR-146a is an important member of the negative feedback loop that controls (TLRs) signaling to NF-κB by its direct targets of TRAF6 and IRAK-1. (3) As such, miR-146a could serve as a key negative regulator of inflammation.

Inflammation and recruitment of leukocytes during epithelial injury are now recognized as major mediators of all phases of endothelial and tubular cell injury during AKI^29^. Lack of miR-146a expression results in hyperresponsiveness of macrophages to LPS and leads to an exaggerated inflammatory response in endotoxin-challenged mice. In contrast, overexpression of miR-146a in monocytes has the opposite effect. Our data revealed the pathological correlation between the expression of miR-146a and proinflammatory cytokines. In our study, those of inflammatory mediators e.g., TNF-α, IL-1β, IL-6; chemokines responsible for neutrophil (MIP-2), macrophage (MCP-1), RANTES (monocytes and T cells), leukocyte adhesion molecules (Icam1) altered, thus promotes interstitial infiltration, all features of I/R injury. These findings are consistent with previous reports in the endotoxin-tolerant THP-1 sepsis cell models^23^.

According to previous cellular experiments, endotoxin tolerance is associated with reduced TLR4-MyD88 complex formation, impairment of TRAF6 and IRAK-1 activity, down-regulation of NF-κB activity, disruption of chromatin remodeling^15^. Promoter analyses have shown that transcriptional up-regulation of miR-146a induced by TLR4 is NF-κB dependent^22^; miR-146a targets the 3′-UTR region of TRAF6 and IRAK-1 by the post-transcriptional silencing^11^,^25^. Above evidence *in vitro* suggests mir-146a is important in controlling TLR signaling in innate immunnity^7^,^24^. However, our results identify a direct functional link between miR-146a, TRAF6 and IRAK-1 accumulation, and tubular damage in mouse tissue *in vivo*, implying that TLR signaling is impaired due to the translation inhibition of miR-146a targeted adaptor kinases during innate immune activation, giving rise to endotoxin tolerance. Consistent findings that miR-146a mediates translational repression of IRAK-1 and protects innate immune tolerance in the neonate intestine has been reported^35^. In current study, miR-146a-mediated down-regulation of TRAF6 and IRAK-1 is time-dependent and underlying molecular mechanisms is not clear. In addition, we also found that miR-146a knockdown didn’t alter its upstream TLR4 expression (Supplementary Figure S3).

Considering the regulation of TRAF6 and IRAK-1 in TLR4/NF-κB signaling and associated inflammation in endotoxin tolerance^36^, we used an anti-miR-146a oligonucleotides approach to block miR-146a and determine the interrelationship between miR-146a and the NF-κB system. We demonstrated that up-regulation of miR-146a in LPS/IR stimulated kidneys was inversely correlated with activation of NF-κB p65/p50, degradation of IκBα. Our results are consistent with previous studies^36^,^37^. Endotoxin tolerance is linked to overexpression of NF-κB, nuclear translocation of p50/p50 homodimers and decreased levels of the active p65/p50 heterodimers^38^,^39^. However, many studies have showed that NF-κB participates in both the generation of proinflammatory mediators and the antiapoptotic pathway, and the balance between these determines the fate of I/R injured kidney cells^40^. Our previous studies confirmed that administration of NF-κB decoy oligodeoxynucleotides *in vivo* and curtailed the production of inflammatory cytokine genes, thereby attenuate kidney I/R injury^41^,^42^. The effects of NF-κB blockade are highly dependent on the time course of inflammation and the experimental conditions. Ulf Panzer *et al.* examined that LPS application induced biphasic renal NF-κB binding activity in wild-type animals with a first peak in the proinflammatory induction phase at 3 and 6 h after LPS injection and a second peak during the resolution period of the inflammatory process at 48 h^40^. In current study, miR-146a expression was inversely correlated with NF-κB activation and histology score. Knockdown of miR-146a caused a stronger nuclear translocation of NF-κB p65 at 6 h and 24 h compared with 48 h after I/R injury in LPS + I/R + anti-miR-146a group. Based on these results, miR-146a is an important member of the negative feedback loop that controls (TLRs) signaling to NF-κB.

Given the importance of microRNAs (miRNAs) in AKI, Godwin *et al.* performed microarray to determine the differentially expressed miRNAs following unilateral renal I/R and miR-146a was one of them^27^. Therefore, we determined whether miR-146a played a role in the development of LPS-induced cross-tolerance against kidney I/R injury. We observed an up-regulation of miR-146a after day3 following IRI and achieved its peak after day5. This result was consistent with the results reported by Godwin^25^. Why knockdown of miR-146a in mice underwent ischemia procedures is not further exacerbate renal injury after reperfusion remains unclear. One plausible explanation is that induction of miR-146a occurred at 6 h after LPS whereas up-regulation of miR-146a by I/R injury occurred at day3 (peaking after day7, Supplementary Figure S1). Therefore, miR-146a induced by LPS is significantly earlier than by I/R injury, suggesting that the protective effect of miR-146a might depend on LPS induction instead of I/R injury.

Multiple microRNA (miR-155, let-7e, miR-98) are involved in development of tolerance^21^. The expression of particular microRNA has been shown to be limited to particular cell types. As a multifactorial process, the kidney protection conferred by LPS pretreatment is likely mediated by several mechanisms. Kang He *et al.* demonstrated LPS exposure prior to ischemia–reperfusion ameliorated AKI in mice (cross-tolerance), which was mediated by LPS-induced NF-κB and hypoxia-inducible factor-2a (HIF-2a) signaling^17^. The current study indicates that TLR4/ NF-κB signaling regulation by miR-146a represents another important mechanism.

In summary, our studies indicate that miR-146a contributes to the establishment of LPS induced cross-tolerance based on the inverse correlation with proinflammatory cytokine production and the repressed protein levels of target genes (IRAK-1and TRAF6) and NF-κB activation. The role of miR-146a in controlling cytokine and TLR signaling is through a negative feedback regulatory loop. Thus, miR-146a may be a viable strategy to prevent acute inflammatory response and improve the outcome of AKI.

## Methods

### Animal models

A warm kidney I/R model was induced in 8-week-old male C57BL/6 mice (Animal Center of Fudan University, shanghai, China) and as described previously^43^. In brief, anesthesia was induced with intraperitoneal sodium pentobarbital (80 mg/kg body weight) and kidney I/R was induced by bilateral kidney pedicle clamping for 30 min, followed by reperfusion for the indicated time. Sham controls underwent all the same surgical procedures except vascular occlusion. This study was approved by the International Animal Care and Use Committee of Fudan University and adhered strictly to the National Institutes of Health Guide For the Care and Use of Laboratory Animals.

### Experimental design

LNA-modified anti-scrambled or anti-miR-146a oligonucleotides (Exiqon, Woburn, MA) were diluted in saline (5 mg/ml), and administered into the tail vein (10 mg/kg) 2 hour prior to LPS injection. At 24 h after exposure to either normal saline (NS) or lipopolysaccharide (LPS, from *E. coli* serotype 055:B5, 3 mg/kg body weight), mice were subjected to kidney ischemia or sham surgery. The animals were divided into following groups (n = 6 per group): (1) I/R + anti-miR-146a + I/R and I/R + scrambled control; (2) LPS + I/R + anti-miR-146a and LPS + I/R + scrambled control; (3) LPS + Sham; (4) NS (normal saline) + Sham. Blood and kidney samples were examined at 6, 24 and 48 h after the surgery. For time course experiments, samples were collected at 0, 1, 2, 3, 5, 7 days following the I/R or sham injury in additional groups of mice (n = 4 per group). Plasma creatinine was measured using the improved Jaffe method (Quantichrom creatinine Assay Kit, BioAssay Systems, Hayward, CA). Concentrations of cytokines in tissue homogenate were examined by commercially available enzymelinked immunosorbent assay (ELISA) kit (R&D Systems, Minneapolis, MN) for interleukin (IL)-1β, and tumor necrosis factor (TNF)-α, according to the manufacturer’s protocol.

### Assessment of kidney injury

Kidneys were fixed in 10% formalin and embedded in paraffin. Histopathological changes were assessed on periodic acid–Schiffstained 4-mm-thick sections in 10 nonoverlapping fields (original magnification x200) of the corticomedullary junction and outer medulla. Tissue damage was assessed in a blinded manner and scored using a tubular damage score, as previously described^44^. Briefly, injury was scored according to the percentage of damaged tubules (loss of brush border, shedding of both necrotic and viable epithelial cells into the tubular lumen, tubular dilation, cast formation, and cell lysis): (i) less than 25% damaged; (ii) 25–50% damaged; (iii) 50–75% damaged; and (iv) more than 75% damaged.

### Immunohistochemistry

Kidney sections from mice were prepared as described A citrate-based antigen retrieval solution was used. Sections were exposed to 3% H_2_O_2_ in methanol for 5 minutes to quench endogenous peroxidases. Endogenous mouse Ig staining was blocked with M.O.M^TM^ mouse IgG blocking regent (Vector Laboratories) for 1 h. Sections were incubated with the CD68 (ED-1, mouse monoclonal; Abcam, Cambridge, MA) or MPO (mouse monoclonal, 1:200; Novus) or anti-P50 (sc-114, rabbit polyclonal 1:5000), anti-cleaved Caspase-3 (#9661, rabbit polyclonal 1:500; cell signaling Technology, Danvers, MA), TLR4 (mouse monoclonal, 1:200; Abcam, Cambridge, MA) overnight at 4 °C for overnight. After washing, sections were incubated with an M.O.M^TM^ biotinylated anti-mouse IgG or anti-rabbit biotinylated secondary antibody at room temperature for 30 min, and then with the avidin–biotin–peroxidase complex (Vector M.O.M.^TM^ Immunodetection Kit, Vector Laboratories). The reaction products were developed using the 3, 3′-diaminobenzidine substrate from Vector Laboratory, mounted with a glass coverslip, and photographed using a Zeiss Axioplan2 microscope with a Q-imaging MP3.3 RTV camera under 200 or 400 magnification. The area labeled was quantified using Image-Pro Plus software.

### TUNEL assay

DeadEnd Colometric TUNEL System from Promega (Madison, WI) was used to detect apoptotic cells on formalin-fixed, paraffinembedded kidney sections. Manufacturer’s protocol was used to processes the sections. The number of TUNEL-positive cells from 10 areas of randomly selected kidney cortex was counted under a light microscope.

### Real-time RT-PCR

Quantitative RT-PCR for gene expression were carried out using cDNA reverse transcribed from RNA extracted from harvested kidney tissue as described using primers corresponding to the gene of interest. Total RNA was extracted using Trizol (Invitrogen, Carlsbad, CA), followed by quantification. Extracted RNA was reverse-transcribed to complementary DNA (PrimeScript RT reagent Kit; TaKaRa, Kyoto, Japan). PCR was performed using SYBR Green Master Mix (Premix Ex TaqTM TaKaRa and the Applied Biosystems 7500 Real-time PCR system). PCR primers were designed using Primer-Blast (NCBI) to span at least one intron of the targeted gene. Ct values of target genes were normalized to GAPDH and presented as fold increase compared to the reference experimental group using the 2−△△^CT^ method. PCR primers used are listed (Table 1).

### RT- and TaqMan-based real-time PCR

Total RNA from kidneys samples was reverse-transcribed using miRNA-specific stemloop RT primers, RTase, RT buffer, dNTPs, and RNase inhibitor according to the manufacturer’s instructions (Applied Biosystems; ABI, Foster City, CA). Expression level of miR-146a was quantified using real-time reverse transcription-PCR with the Taqman chemistry (ABI) and miRNA-specific TaqMan primers (Table 1), as described previously^45^. U6 small nuclear RNA was used as endogenous housekeeping control for data normalization of miRNA levels.

### Western blot analysis

The kidneys were lysed with RIPA solution containing 1% NP40, 0.1% SDS, 100 mg/ml PMSF, Complete Protease Inhibitor Cocktail Tablets (Roche, Indiana, USA) on ice. Nuclear extracts were isolated from harvested kidney medulla using NE-PER Nuclear and Cytoplasmic Extraction Reagents (Pierce Biotechnology, Inc, USA). The purity of nuclear proteins was verified by western blot analysis to confirm the absence of β-actin (cytosolic protein) and the presence of Histone H3 (nuclear protein). Equal amounts of protein samples were electrophoretically separated on sodium dodecyl sulfate polyacrylamide gel, transferred to polyvinylidene fluoride membranes (Millipore), and probed with primary antibodies as follows: anti-BcL-xL (#2764, rabbit monoclonal 1:1000), anti-p65 (#6956, mouse monoclonal 1:1000) and anti-phosphorylated P65 (#3036, mouse monoclonal 1:1000) were from Cell Signaling Technology (Danvers, MA); anti-TRAF6 (sc-7221, rabbit polyclonal 1:1000) anti-IRAK-1(sc-7883, rabbit polyclonal 1:1000), anti-IκB-α (sc-371, rabbit polyclonal 1:1000), anti- β-actin(sc-8432, mouse monoclonal 1:1000), GAPDH (sc-365062, mouse monoclonal 1:1000) were from Santa Cruz Biotechnology (Santa Cruz, CA); Histone H3 (ab1791, rabbit polyclonal 1:1000) was from abcam. Then the membranes were incubated with a horseradish peroxidase-conjugated secondary antibody, and developed by chemiluminescent Horseradish Peroxidase Substrate (Millipore, Billerica, MA). All values were normalized to the loading control and expressed as fold increase relative to control.

### Statistical analysis

Data are expressed as mean ± SEM. For comparison of means with more than two subgroups, the nonparametric Kruskal-Wallis ANOVA followed by Dunnett with significance cut off <0.05 was used. All analyses were performed using GraphPad Prism Software.

## Additional Information

**How to cite this article**: Dai, Y. *et al.* miR-146a is essential for lipopolysaccharide (LPS)-induced cross-tolerance against kidney ischemia/reperfusion injury in mice. *Sci. Rep.* **6**, 27091; doi: 10.1038/srep27091 (2016).

## Supplementary Material

Supplementary Information

## Figures and Tables

**Table 1:** Real-time PCR primers and miR-146a TaqMan primers.

Gene	Forward	Reverse
IRAK1	5′-GAGACCCTTGCTGGTCAGAG-3′	5′-GCTACACCCACCCACAGAGT-3′
TRAF6	5′-GCCCAGGCTGTTCATAATGT-3′	5′-CGGATCTGATGGTCCTGTCT-3′
IL-6	5′-CCTCTCTGCAAGAGACTTCCATCCA-3′	5′-AGCCTCCGACTTGTGAAGTGGT-3'
MCP-1	5′-TTAAAAACCTGGATCGGAACCAA-3′	5′-GCATTAGCTTCAGATTTACGGGT-3′
RANTES	5′-GAGTGACAAACACGACTGCAAGAT-3′	5′-CTGCTTTGCCTACCTCTCCCT-3′
MIP-2	5′-GCCCCCAGGACCCCA-3′	5‘-CTTTTTGACCGCCCTTGAGA-3′
ICAM-1	5′-GTGATGCTCAGGTATCCATCCA-3′	5′-CACAGTTCTCAAAGCACAGCG-3′
IL-1β	5′-CCTTCCAGGATGAGGACATGA-3'	5′-TCATCCCATGAGTCACAGAGGAT-3′
TNF-α	5′-TTCTGTCTACTGAACTTCGGGGTGATCGGTCC-3′	5′-GTATGAGATAGCAAATCGGCTGACGGTGTGGG-3′
Il-10	5′-GCCTTCAGTATAAAAGGGGGACC-3′	5′-GTGGGTGCAGTTATTGTCTTCCCG-3′
GAPDH	5′-GCCATCAACGACCCCTTCAT-3′	5′-ATGATGACCCGTTTGGCTCC-3′
Gene name	MiRBaseID	Sequences
mmu-miR-146a	MIMAT0000158	UGAGAACUGAAUUCCAUGGGUU
