# Supplementary material for: miR-146a is essential for lipopolysaccharide (LPS)-induced cross-tolerance against kidney ischemia/reperfusion injury in mice
Source: Sci Rep. 2016 Jun 2;6:27091. doi: 10.1038/srep27091 (PMC4890025; doi:10.1038/srep27091)

Supplementary Information

Title: miR-146a is essential for lipopolysaccharide (LPS)-induced cross-tolerance against kidney ischemia/reperfusion injury in mice.

Authors and Affiliations: Yan Dai [1,2,3,6], Ping Jia [1,2,3,6], Yi Fang[1], Hong Liu [1], Xiaoyan Jiao [1,2,3], John C. He [4, 5], Xiaoqiang Ding* [1,2,3]. [1]Division of Nephrology, Zhongshan Hospital, Fudan University, Shanghai, China; [2]Kidney and Dialysis Institute of Shanghai, Shanghai, China; [3]Kidney and Blood Purification Laboratory of Shanghai, Shanghai, China; [4]Department of Medicine/Nephrology, Icahn School of Medicine at Mount Sinai, New York, New York, USA; [5]Kidney Section, James J Peter Veteran Administration Medical Center at Bronx, NY, United States. [6]These authors contributed equally to this work. Correspondence and requests for materials should be addressed to X.D. (E-mail: ding.xiaoqiang@zs-hospital.sh.cn)

Supplementary S1: (a) Time course of miR-146a and miR-21 levels between kidney I/R injury and sham controls groups. miR-146a and miR-21 was examined and normalized to U6 by TaqMan-based real-time PCR, fold changes were calculated against the mean value of Sham group at each time point. (n=3 mice per each time point.*P<0.05,**P < 0.01,$^{\#}P$<0.001,vs. Sham group, respectively). (b) Proinflammatory cytokine mRNA profile in mice at 24h after kidney I/R surgery with LNA-anti-miR-146a treatment. LNA-anti-miR-146a and LNA anti-scrambled oligonucleotide were administered 24h before I/R surgery. (c) Representative periodic acid–Schiff (PAS)-stained renal sections from mice treated with either I/R+Scrambled control and I/R+anti-miR-146a procedures (original magnification ×200). (d) Semiquantitative analysis of tubular damage in I/R+Scrambled control and I/R+anti-miR-146a mouse kidney at 24h after reperfusion. ($^{\#}P$<0.001vs. I/R+ Scrambled control).

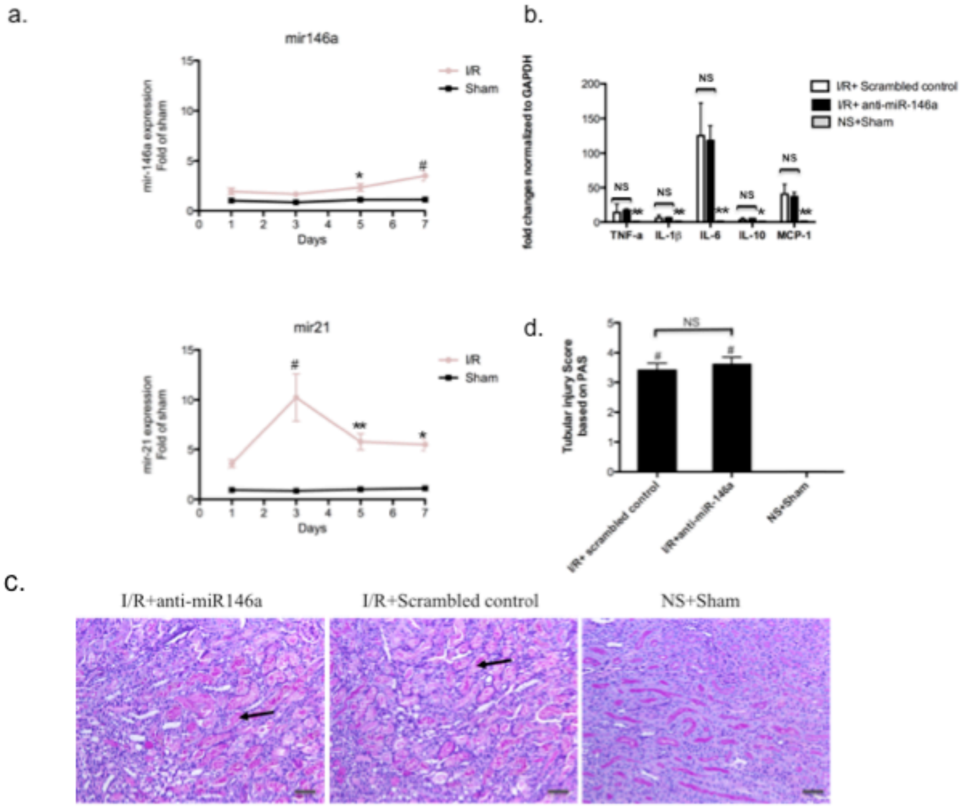

Supplementary S2 (a) The expression of phosphorylated p65 (p-p65) (nuclear extracts), and IkBα, BcL-xL, T-P65 (cytosolic extracts) in the 6h groups was confirmed by western blot analysis. Co-detection of Histone H3 (nuclear) and β-actin (cytoplasm) were performed to assess equal loading. (b) Western blots from three experiments were quantified by densitometry analysis. The ratios of phosphor-protein to total protein for p65 were calculated. The fold changes relative to LPS+Sham protein are shown. (n=3, $^{\#}P<0.001$, $^{**}P<0.01$,vs. LPS+I/R+Scrambled control group).(c) The expression of phosphorylated p65 (p-p65) (nuclear extracts), and IkBα, BcL-xL, T-P65 (cytosolic extracts) in the 48h groups were confirmed by western blot analysis.(d) The Western blots from three experiments were quantified by densitometry analysis. The ratios of phosphor-protein to total protein for p65 were calculated. The fold changes relative to LPS+Sham protein are shown. (n=3, $^{**}P<0.01$,$^{*}P<0.05$ vs. LPS+I/R+Scrambled control group)

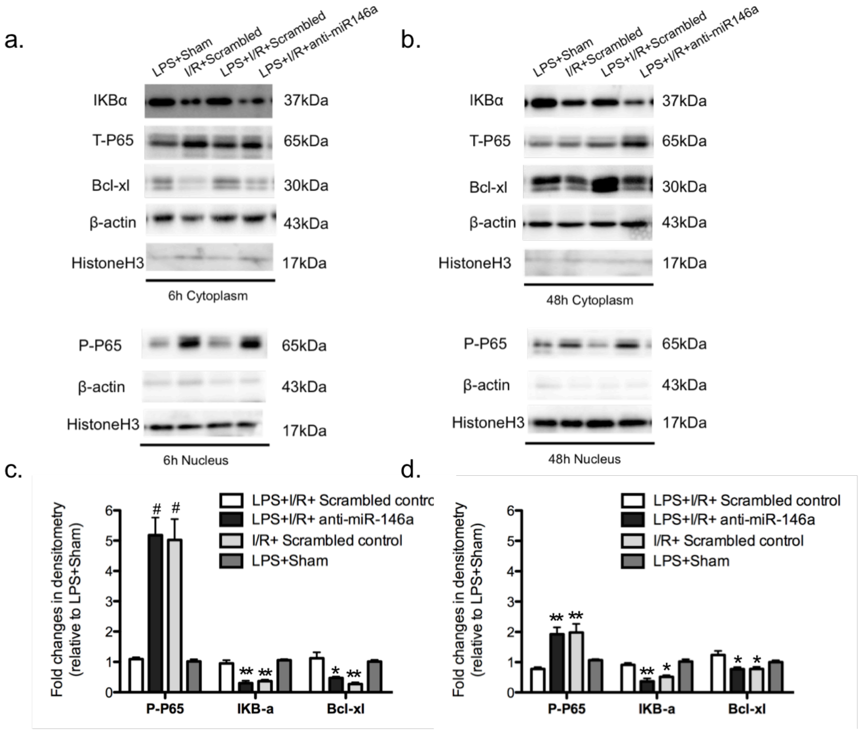

Supplementary S3: (a) Representative kidney sections immunostained for TLR4 (original magnification ×200 and ×400, Bar=50um, the black arrow indicates positive area). TLR4 was expressed by tubular cells and displayed a similar marked elevation in mice receiving anti-miR-146a or anti-scrambled oligonucleotides at 24h after I/R injury. (b) TLR4 expression was scored in mouse kidney. (n=6mice per group, *P<0.05, vs. LPS+I/R+Scrambled control).

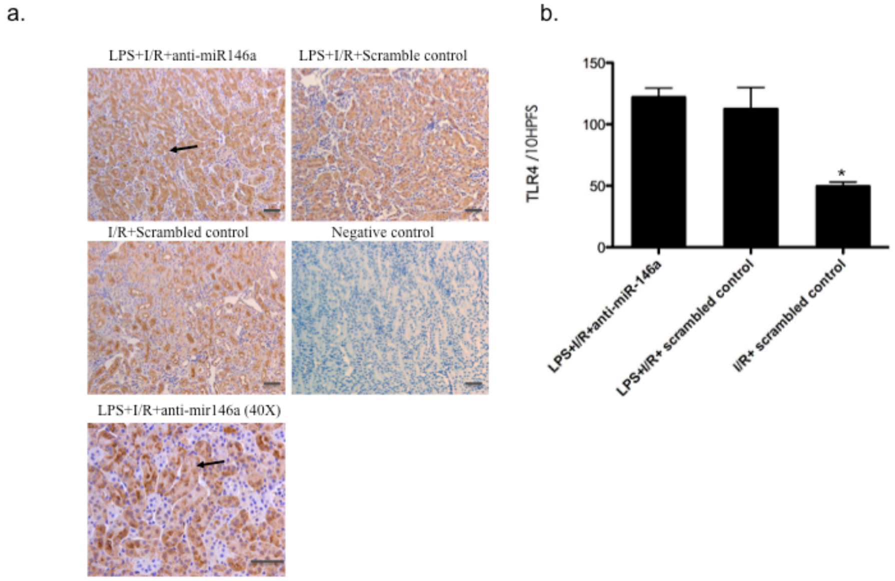

Supplement: Supplementary Information [file srep27091-s1.pdf]
